# Efficacy of lidocaine in patients receiving palliative care with opioid-refractory cancer pain with a neuropathic component: study protocol for a randomized controlled study

**DOI:** 10.1186/1745-6215-15-318

**Published:** 2014-08-12

**Authors:** Sébastien Salas, Pascal Auquier, Florence Duffaud, Stéphanie Ranque Garnier, Mélanie Deschamps, Stéphane Honoré, Patrick Sudour, Karine Baumstarck

**Affiliations:** Service d’Oncologie Médicale Adulte et de Soins Palliatifs, Centre Hospitalier Universitaire Timone, AP-HM, Aix-Marseille University, 27 bd Jean Moulin, 13385 Marseille, France; CRO2, INSERM U911, Aix-Marseille University, 27 bd Jean Moulin, 13385 Marseille, France; EA3279, Self-perceived Health Assessment Research Unit, School of Medicine, Université de la Méditerranée, 27 bd Jean Moulin, Marseille cedex 05, F-13385 France; Unité d’Expertise Pharmaceutique et Recherche Biomédicale, Pôle Pharmacie, AP-HM, 27 bd Jean MOULIN, 13385 Marseille, France; Direction de la Recherche, AP-HM, 80 rue Brochier, 13005 Marseille, France

**Keywords:** Lidocaine, Randomized controlled trial, Opioid-refractory cancer pain, Palliative care

## Abstract

**Background:**

The management of patients suffering from opioid-refractory cancer pain with a neuropathic component remains an important challenge for healthcare workers. Only one retrospective study specifically reported the use of intravenous (IV) lidocaine amongst the palliative care unit population, the study found that there was a positive response to this therapy. These preliminary uncontrolled results need to be confirmed by randomized controlled trials. The primary objective of this study is to assess the analgesic efficacy of IV lidocaine in patients in palliative care suffering from opioid-refractory cancer pain with a neuropathic component. The secondary objectives are to assess the tolerance of, symptomatology, and patient satisfaction with the therapeutic approach.

**Methods/Design:**

This will be a multicenter, prospective, randomized, placebo-controlled, double-blind, two-parallel group study. It will take place in eight adult palliative care units across France. The main inclusion criteria are as follows: adult patients suffering from opioid-refractory cancer pain with a neuropathic component, and those receiving palliative care as defined by French Society of Palliative and Support Care. Participants will be randomized (1:1 allocation ratio) to one of two treatment groups: a) lidocaine-experimental group (intravenous lidocaine), or b) placebo-control group (intravenous saline solution). Evaluation assessments will be taken at baseline (T0 randomization), 40 minutes (T1), 120 minutes (T2), 12 hours (T3), 24 hours (T4), 48 hours (T5), and 14 days (T6) after baseline. The primary endpoint is change in the pain level between T0 and T1. The secondary endpoints are: changes in the pain level between T0 and other times, intensity of the neuropathic pain component, daily opioid consumption, symptoms (as classified by the MD Anderson Symptom Inventory), adverse events, and patient’s satisfaction (measured using the Pain Treatment Satisfaction Scale). A sample size of 200 individuals will be needed to obtain 90% power to detect a 25% difference in pain success at T1 between the two groups; pain success is classified as a 30% decrease in the pain level between T0 and T1 (10% of patients lost to follow-up expected).

**Discussion:**

The randomized, double-blind, placebo-controlled design is the most appropriate design to demonstrate the efficacy of a new experimental intervention (Evidence-Based Medicine Working Group classification). National and international recommendations could be updated based on the findings of this study.

**Trial registration:**

Current controlled trials NCT02137954 (registration date: 7 May 2014).

## Background

The management of patients suffering from pain that is refractory to opioids remains an important challenge for healthcare workers. Patients experiencing pain with a neuropathic component, those experiencing an isolated neuropathic component, and those experiencing mixed pain including neuropathic and nociceptive components, are of particular concern. Some therapies such as tricyclic antidepressants, anticonvulsants, or antiarrhythmics, have already demonstrated efficacy but may require a few weeks to be fully effective. Analgesic interventions with more rapid action would be valuable.

The analgesic effect of intravenous (IV) sodium channel blockers, such as lidocaine, could be a viable alternative as they suppress the ectopic discharges recorded in a neuroma [1]. However, some experimental studies performed on animals and humans have also provided evidence of a central action of lidocaine, possibly through N-methyl-D-aspartate (NMDA) receptor binding [2]. Even if central neurological (generalized seizures and convulsive events) and cardiovascular adverse effects are well-documented with IV lidocaine, low doses do not cause significant hemodynamic changes.

Previous randomized controlled trials (RCTs) have already assessed the efficacy of IV lidocaine on non-cancer neuropathic pain such as diabetic neuropathy [3, 4], postherpetic neuralgia [5, 6], spinal cord injury [7], peripheral nerve injury [8, 9], post-amputation pain [10], sciatica [11], and neuralgia [12]. In neuropathic cancer pain only three randomized placebo-controlled studies reported the use of IV lidocaine [13–15], with discordant findings. Two did not find any significant difference in pain between the lidocaine and placebo groups, but those studies were performed on a small sample (10 patients in each trial) [13, 14]. The third, most recent study, which was performed on a larger sample (n = 50), demonstrated a significant analgesic effect of IV lidocaine compared with the placebo [15].

Only one retrospective study specifically evaluated the needs of the palliative care unit population [16]. The study found that among 82 patients admitted to palliative care and suffering from opioid-refractory cancer pain, 91% had a major response to parenteral lidocaine. These preliminary uncontrolled results need to be confirmed by RCTs.

Since 2010, despite a limited amount of evidence, the French drug and device regulation agency (Agence Nationale de Sécurité du Médicament, ANSM) has recommended local anesthetics given parenterally for palliative care. Lidocaine can be used to treat cancer pain that is refractory to standard opiates in the hospital at the medical staff’s discretion. The maximum dose is 8 mg/kg per day. Monitoring for toxicity should be implemented, specifically for the occurrence of a metallic taste in the mouth, numbness of the lips and tongue, hot and/or cold feelings, and headache leading to lidocaine discontinuation.

These observations prompted us to establish a multicenter, prospective, two-group, placebo-controlled, double-blind, randomized study with the primary objective of assessing the analgesic efficacy of IV lidocaine in patients in palliative care suffering from opioid-refractory cancer pain with a neuropathic component. The secondary objectives are to assess the tolerance of, symptomatology, and patient satisfaction with this therapeutic approach.

## Methods/Design

### Design

This multicenter, prospective, randomized, placebo-controlled, double-blind, two-parallel group study is performed to compare the efficacy of the use of lidocaine (experimental group) with the use of a placebo (control group). The study protocol was designed using the recommendations of the Consolidated Standards of Reporting Trials (CONSORT) statement.

### Partners

The sponsor of the study is the Assistance Publique-Hôpitaux de Marseille (AP-HM, France). The recruiting will be performed in eight adult palliative care units across France. The methodological support will be provided by the Clinical Research Unit of Palliative Care (Unité Aide Méthodologique de Soins Palliatifs, AP-HM, France), the Clinical Investigation Unit (Centre d’Investigation Clinique, AP-HM, France), and the Self-perceived Health Assessment Research Unit (Aix-Marseille University, Marseille, France). The central pharmacy of AP-HM is in charge of the assignment, allocation, and delivery of the treatments. This work is supported by institutional grants from the French 2012 National Program of Clinical Research (Programme Hospitalier de Recherche Clinique National). All details are provided in Table 1.

**Table 1:** **French partners**

Oncologists	Centers of enrollment
Pr Sébastien Salas	Coordinating investigator
	PCU, public academic teaching hospital Timone, Marseille
Pr Régis Aubry	PCU, public academic teaching hospital, Besançon
Dr Sophie Bayle	MCU, public academic teaching hospital, Saint-Etienne
Dr Benoit Burucoa	PCU, public academic teaching hospital, Bordeaux
Dr Bénédicte De Corbière	PCU, public academic teaching hospital, Beaujon, Paris
Dr Virginie Guastella	PCU, public academic teaching hospital, Clermont Ferrand
Dr Guillemette Laval	PCU, public academic teaching hospital, Grenoble
Dr Pascale Vassal	PCU, public academic teaching hospital, Saint-Etienne
**Methodologists**	
Pr Pascal Auquier	Public health, public academic teaching hospital Nord, Marseille
Dr Karine Baumstarck	Clinical research unit, public academic teaching hospital, Marseille
Dr Stephanie Ranque	Clinical research unit, public academic teaching hospital, Marseille

MCU medical care unit including specific palliative care places; PCU palliative care unit.

### Participants

The details of the inclusion and exclusion criteria are provided in Table 2. The main inclusion criteria are patients suffering from cancer pain (neuropathic or mixed) that is refractory to standard opiates, receiving palliative care as defined by French Society of Palliative and Support Care and receiving or not receiving orally or intravenously morphine or oxycodone. The main exclusion criteria are patients with a known contraindication to the use of lidocaine, with altered sleepiness, or with impaired cognitive function.

**Table 2:** **Selection criteria**

Inclusion criteria	
- Patient aged 18 years or older	
- Patient suffering from cancer pain refractory to standard opiates (numeric pain intensity scale (NPIS) ≥4/10 after 24 hours of continuous intravenous morphine or oxycodone administration [17], analgesics drugs for adults cancer nociceptive pain]), regardless of the nature of the primary cancer	
- Patient suffering from cancer neuropathic or mixed pain (DNA survey score ≥4 [18])	
- Patient receiving palliative care as defined by French Society of Palliative and Support Care [Charte des Soins Palliatifs, 1996, Act No. 99–477 of 9 June 1999 to guarantee the right of access to palliative care] according to the definition of the World Health Organization (WHO)	
- Patient receiving or not receiving orally or intravenously morphine or oxycodone	
- Patient with a histological diagnosis of cancer (locally advanced or metastatic disease)	
- Patient without curative cancer treatment, and with or without palliative anticancer treatment	
- atient receiving tricyclic antidepressants, anticonvulsants, or antiarrhythmics for less than two weeks	
- Patient hospitalized in a specific palliative care unit	
- Patient with an estimated survival of more than 48 hours (physician estimation)	
- Written informed consent for participation obtained prior to any study procedures.	
**Exclusion criteria**	
- Patient with a known hypersensitivity to lidocaine	
- Patient with a history of porphyria, arrhythmias, disorders of atrioventricular conduction requiring permanent pacing not yet realized, uncontrolled epilepsy, or uncontrolled hypertension	
- Patient with hematologic malignancy, abnormal renal, hepatic, and cardiac functions	
- Patient with an altered sleepiness (Epworth scale score ≤16)	
- Patient with altered cognitive function (Test ELEmentaire de Concentration, Orientation et Mémoire score >11)	
- Patient not native French speaker	
- Patient defined as a vulnerable subject (minor subject, pregnant or nursing woman, subject who is freedom-deprived)	

### Treatments

The administration of the treatment (lidocaine or placebo) will be blinded and neither the patient nor the nurse responsible for the administration will be informed of the nature of the treatment. The treatment (lidocaine or placebo) will be administered during 48 hours.

#### Morphine hydrochloride or oxycodone administration

The protocol for administration of first-line level 3 opiates (morphine hydrochloride or oxycodone) will be given to all the included patients. This protocol will be conducted according to the recommendations of the World Health Organization (WHO), the ’Standard Options and Recommendations (SOR) on cancer nociceptive pain treatments for adult patients’ published by the French Union of Comprehensive Cancer Centers (Fédération nationale des centres de lutte contre le cancer, FNCLCC), and the European Association for Palliative Care (EAPC) recommendations on morphine and alternative opioids in cancer pain [17]. Although IV morphine hydrochloride or oxycodone administration is generally restricted to patients for whom oral administration is impractical, the included patients will be systematically intravenously treated to standardize the therapeutic protocol and for quicker action. For patients already taking oral opiates, the IV dose for 24 hours will be equal to one-third of the oral dose for morphine hydrochloride and two-thirds of the oral dose for oxycodone. For patients without initial oral opiate treatment, the loading dose will be 1 mg/kg per day oral equivalent, except in the setting of renal insufficiency or in the elderly, in which case the dose will be 0.25 to 0.5 mg/kg per day. There will be no maximum dose as long as the side effects can be controlled. The daily morphine or oxycodone dose may be increased daily by 50% if necessary. Morphine interdoses will be planned at one tenth of the daily dose. The interval between interdoses will be fixed at one hour [17].

#### Lidocaine - experimental group

Lidocaine will be administered to participants randomized to the experimental group. The lidocaine will be administered by continuous IV infusion. The initial dose will be 5 mg/kg per day for the first 24 hours and then 8 mg/kg per day after 24 hours if the pain level does not decrease by at least 30% between the time of administration and the following 24 hours.

#### Placebo - control group

An IV saline solution (placebo) will be administered to participants randomized to the control group. The protocol for administration of the placebo will be strictly similar to the lidocaine protocol and the protocol for administration of morphine hydrochloride or oxycodone will be the same as in the experimental group. The placebo will be administered by continuous IV infusion throughout the treatment period.

#### Other treatments

No other opioids will be added during the study period. Tricyclic antidepressants, anticonvulsants, and antiarrhythmics may be given. Support care and symptomatic treatments will be delivered in accordance with the French SOR [17].

#### Monitoring during treatment

Any adverse effects of the lidocaine treatment such as a metallic taste, perioral numbness and tingling, feeling hot and cold, headache, and tremors, will be cautiously evaluated to determine whether the IV lidocaine should be interrupted. The patients will be carefully monitored by electrocardiograph for 24 hours after the initiation of the treatment.

### Recruitment and follow-up

#### Inclusion

The participating centers will be used to recruit the patients. Eligible patients satisfying the screening inclusion and exclusion criteria will be randomized into one of the two treatment groups after completing the consent form.

#### Randomization

Computer-generated randomized lists will be drawn up before the beginning of the study, using a permuted block design, under the responsibility of the clinical research unit (AP-HM). The randomization will be stratified by center and the nature of the opioid (morphine hydrochloride or oxycodone). The patients will be randomized to one of the two treatment groups using a computer-generated randomization schedule with a 1:1 allocation ratio. The randomization will be to either the experimental group (IV morphine hydrochloride or oxycodone in addition to lidocaine) or the control group (IV morphine hydrochloride or oxycodone in addition to placebo). Only the pharmacist will know the allocation. Prior to and during the treatment period, the participant, treating medical staff, and the investigators will all be unaware of the allocation.

#### Follow-up and data collection

The evaluation will be performed at seven different time points: baseline (T0 after randomization and before starting lidocaine or placebo introduction) and 40 minutes (T1), 120 minutes (T2), 12 hours (T3), 24 hours (T4), 48 hours (T5), and 14 days (T6) after baseline. The study procedure and data collection are detailed in Table 3.

**Table 3:** **Study procedure**

	T0	T1, T2, T3	T4, T5	T6
	Inclusion	Administration	Post-administration	Day-14
Consent	x			
Randomization	x			
Clinical examination	x			
NPIS	x	x	x	x
NPSI	x	x	x	x
ECG	x	x		
Tolerance		x		
Opioid consumption		x	x	x
MDASI	x		x	
Adverse events		x	x	x
Intercurrent events		x	x	x
Satisfaction PTSS	x		x	x

ECG, electrocardiograph; MDASI, MD Anderson Symptom Inventory; NPIS, Numeric Pain Intensity Scale; NPSI, Neuropathic Pain Symptom Inventory; PTSS, Pain Treatment Satisfaction Scale.

### Endpoints

#### Primary endpoint

Analgesic efficacy will be assessed by several endpoints. The primary endpoint will be defined as the change in the pain level between baseline (T0) and 40 minutes (T1) after baseline. Pain level will be assessed using a self-administered numeric pain intensity scale (NPIS), ranging from 0 (no pain) to 10 (worst pain possible). A minimum 30% decrease in the pain level between the baseline and T1 will define the success of the treatment, and all other cases will be classed as failure of the treatment.

#### Secondary endpoints

Analgesic efficacy will be assessed by the following endpoints: the changes in the pain level (as measured by the NPIS) between T0 and T2, T3, T4, T5, and T6; the intensity of the neuropathic pain component as measured by the Neuropathic Pain Symptom Inventory (NPSI) at T4, T5, and T6 [18]; the NPSI is a 12-item self-administered questionnaire describing the intensity of the symptoms associated with neuropathic pain;raw values of the pain levels (as measured by NPIS) at each evaluation time and the percentage of the reduction between the initial level of pain and the other evaluation times;daily opioid consumption during the study period (morphine or oxycodone).

Symptoms will be assessed using the French version of the MD Anderson Symptom Inventory (MDASI). The MDASI is a 19-item self-reported symptom scale largely used in palliative care patients [19, 20].

The tolerance to the treatments (morphine-oxycodone and lidocaine) will be assessed during the first 48 hours following the treatment administration. Adverse events will be cautiously observed (intensity and discomfort). For morphine and oxycodone, the following symptoms will be monitored: nausea and/or vomiting, constipation, sleepiness, psychodysleptic effects, and signs of opioid overdose. For lidocaine, the following symptoms will be monitored: arrhythmia, blurred vision, headache, malaise, tremors, metallic taste, nausea and/or vomiting, perioral numbness and tingling, sedation, and tinnitus.

Patient satisfaction will be assessed using the Pain Treatment Satisfaction Scale (PTSS) [21, 22], which includes 39 items grouped into five categories: information, medical care, impact of current pain medication, satisfaction with pain medication, and side effects. A score will be calculated for each category between 0 (lowest level of satisfaction) and 100 (highest).

### Pharmaceutical aspects

All study drugs will be packaged in blinded trial packs by a clinical trial pharmacist who will be blinded to the interventions and outcomes. Lidocaine, oxycodone, and morphine are controlled drugs.

Standard operating procedures (SOPs) have been developed for every stage of a controlled drug’s journey from procurement (ordering, receipt, and transport) to safe storage, supply, administration, destruction, and for dealing with an adverse event. The SOPs will be accessible to the staff at all times.

### Statistical considerations

#### Sample size, power, and statistical methods

The sample size was determined to obtain 90% power to detect a 25% difference in pain success at 40 minutes between the two groups, as this difference is considered to be clinically significant. Pain success was defined as a 30% decrease in the pain level between baseline and 40 minutes. In accordance with a previous study [15], we hypothesize that pain success for 50% of the patients in the lidocaine experimental group, and 25% of the patients in the placebo control group will be required to be classified as successful. With the threshold for statistical significance set at a *P* value of 0.05 (two-sided alpha), these calculations show that 170 patients are needed (85 per group). Assuming that a potential 10% of patients will be lost to follow-up, a total of 200 individuals need to be included.

#### Data analysis

The data will be analyzed using SPSS Statistics for Windows, Version 17.0. Chicago, USA. The patients found to be eligible but not included in the study will be described and compared with the included patients. The patients who present at least one of the following conditions will be not in the final analysis: patients inappropriately included despite not providing consent and patients who withdraw their consent. The full analysis population (including all subjects who will be randomized and will be at least evaluated at T1) will be used in the primary analysis, and the per protocol population (including all subjects who will be randomized and will not have major protocol deviations) will be used in the secondary analysis to assess the robustness of the results. No interim analysis is planned. The normality of these parameters will be estimated using frequency histograms and the Shapiro test. The baseline parameters will be compared between the two groups (‘control’ and ‘experimental’) using the chi^2^ test or Fisher’s exact test for categorical variables and Student’s t-test for continuous variables.

The analysis of the primary endpoint will consider the proportion of pain success (patients with a 30% reduction in their pain level at T2, as measured using the NPIS) calculated for each group. These proportions will be compared using the chi^2^ test or Fisher’s exact test for categorical variables. Changes in the pain level between each baseline score and the score at T2, T3, and T4 will be compared between the two groups, and the analysis of variance for repeated measurements will be performed to compare the changes in the pain scores over time between the two groups. The NPSI, MDASI, and PTSS scores will be compared between the two groups using Student’s t-test for continuous variables if applicable (or using nonparametric tests and the Mann–Whitney test). Multivariate analysis using logistic regression models will be performed to determine variables potentially linked to pain success. Variables relevant to the models will be selected based on their clinical significance (specifically the administration of tricyclic antidepressants and/or anticonvulsants and/or antia rrhythmics) and/or a threshold of *P* ≤0.2 in the univariate analysis. The final models will estimate the odd ratios and 95% confidence intervals. All of the tests will be two-tailed with a 5% significance level.

### Ethical aspects, laws and regulations

The study will be conducted in accordance with the Helsinki declaration and the French laws and regulations (Code de la Santé Publique, article L.1121-1/Loi de Santé Publique n°2004-806 du 9 août 2004 relative à la politique de santé publique et ses décrets d’application du 27 août 2006) and the International Conference on Harmonization (ICH) E6 Guideline for Good Clinical Practice. Regulatory monitoring will be performed by the sponsor. The sponsor obtained the approval of the French authorities, including the French ethics committee (Comité de Protection des Personnes Sud Méditerranée V, reference number 14.007) and the French drug and device regulation agency (Agence Nationale de Sécurité du Médicament, reference number 140047A-32), before beginning the study. The ClinicalTrials.gov identifier is NCT02137954. Informed consent will be obtained from all subjects.

## Discussion

To date, no randomized study has determined the analgesic efficacy and tolerance of lidocaine in patients receiving palliative care with opioid-refractory cancer pain with a neuropathic component.

We will use a randomized, double-blind, placebo-controlled design, which is the most appropriate design to demonstrate the efficacy of a new experimental intervention in accordance with the Levels of Evidence classification of the Evidence-Based Medicine Working Group. In the future, national and international recommendations could be updated based on our findings.

However, some issues related to the content of the protocol study should be discussed. A parallel design has been adopted, although an intra-individual crossover design is generally considered to be the most appropriate to assess the efficacy of a new analgesic strategy because of the well-known patient variation in the subjective perception of pain. In the crossover study, each subject acts as his or her own control. Several limitations of crossover trials have led us to avoid this design [23]. The crossover design is suitable for patients in a stable condition, which is not the case for patients with opioid-refractory cancer pain. We further suspect that the treatment may have carryover effects and alter the response to subsequent treatments and that subjects may not be in a comparable condition at the start of each treatment period. Similarly, the introduction of a washout (no treatment) period between consecutive treatments may be problematic because of ethical considerations. Lastly, the design does not allow for the assessment of long-term efficacy [15].

The treatment procedure has been established in accordance with common recommendations. The administration modalities and doses for level III opioids (morphine and oxycodone) are described in the WHO’s pain ladder and in the French recommendations [17]. Lidocaine will be administered according to the French drug and device regulation agency (Agence Nationale de Sécurité du Médicament) [24]; the initial dose of the infusion was arbitrarily designated by the investigators because no consensus was available from the literature [15].

In clinical trials, pain level is usually assessed using the self-administered NPIS [15, 25] and this allows for different endpoints to be discussed. Sharma *et al*. [15] designated the primary endpoint as the proportion of patients with a 50% reduction in their pain level at 2 hours after the beginning of the treatment infusion, but we opted for the proportion of patients with a 30% reduction in their pain level at 40 minutes because of the half-life of the molecule. In addition, the long-term effect of lidocaine will be confounded because the patients will undergo different interventions after the study period.

The selection criteria included cancer patients receiving palliative care with opioid-refractory pain with a neuropathic component to ensure a homogeneous population, but it can be assumed that patients with opioid-refractory non-cancer pain could also benefit from this therapy.

The randomization could have been stratified by other factors (baseline level of pain, type of pain (pure versus mixed neuropathic), analgesic dose equivalents, or co-administration of non-opioid agents, and so on), but this was not done due to the large number of other available factors.

## Trial status

At the time of manuscript submission (2014, July), the status of the trial is ‘not yet recruiting’.

## Abbreviations

ANSM: Agence Nationale de Sécurité du Médicament
AP-HM: Assistance Publique-Hôpitaux de Marseille
CONSORT: Consolidated standards of reporting trials
EAPC: European association for palliative care
FNCLCC: Fédération nationale des centres de lutte contre le cancer
ICH: International conference on harmonization
IV: Intravenous
MDASI: MD Anderson symptom inventory
NMDA: N-methyl-D-aspartate
NPIS: Numeric pain intensity scale
NPSI: Neuropathic pain symptom inventory
PTSS: Pain treatment satisfaction scale
RCT: Randomized controlled trial
SOP: Standard operating procedure
SOR: Standard, options and recommendations
WHO: World health organization.

## References

[1] Tanelian DL, Brose WG (1991). Neuropathic pain can be relieved by drugs that are use-dependent sodium channel blockers: lidocaine, carbamazepine, and mexiletine. Anesthesiology.

[2] Biella G, Sotgiu ML (1993). Central effects of systemic lidocaine mediated by glycine spinal receptors: an iontophoretic study in the rat spinal cord. Brain Res.

[3] Kastrup J, Petersen P, Dejgard A, Angelo HR, Hilsted J (1987). Intravenous lidocaine infusion–a new treatment of chronic painful diabetic neuropathy?. Pain.

[4] Viola V, Newnham HH, Simpson RW (2006). Treatment of intractable painful diabetic neuropathy with intravenous lignocaine. J Diabetes Complications.

[5] Rowbotham MC, Reisner-Keller LA, Fields HL (1991). Both intravenous lidocaine and morphine reduce the pain of postherpetic neuralgia. Neurology.

[6] Baranowski AP, De Courcey J, Bonello E (1999). A trial of intravenous lidocaine on the pain and allodynia of postherpetic neuralgia. J Pain Symptom Manage.

[7] Finnerup NB, Biering-Sorensen F, Johannesen IL, Terkelsen AJ, Juhl GI, Kristensen AD, Sindrup SH, Bach FW, Jensen TS (2005). Intravenous lidocaine relieves spinal cord injury pain: a randomized controlled trial. Anesthesiology.

[8] Attal N, Rouaud J, Brasseur L, Chauvin M, Bouhassira D (2004). Systemic lidocaine in pain due to peripheral nerve injury and predictors of response. Neurology.

[9] Wallace MS, Dyck JB, Rossi SS, Yaksh TL (1996). Computer-controlled lidocaine infusion for the evaluation of neuropathic pain after peripheral nerve injury. Pain.

[10] Wu CL, Tella P, Staats PS, Vaslav R, Kazim DA, Wesselmann U, Raja SN (2002). Analgesic effects of intravenous lidocaine and morphine on postamputation pain: a randomized double-blind, active placebo-controlled, crossover trial. Anesthesiology.

[11] Medrik-Goldberg T, Lifschitz D, Pud D, Adler R, Eisenberg E (1999). Intravenous lidocaine, amantadine, and placebo in the treatment of sciatica: a double-blind, randomized, controlled study. Reg Anesth Pain Med.

[12] Marchettini P, Lacerenza M, Marangoni C, Pellegata G, Sotgiu ML, Smirne S (1992). Lidocaine test in neuralgia. Pain.

[13] Bruera E, Kuehn N, Miller MJ, Selmser P, Macmillan K (1991). The Edmonton symptom assessment system (ESAS): a simple method for the assessment of palliative care patients. J Palliat Care.

[14] Ellemann K, Sjogren P, Banning AM, Jensen TS, Smith T, Geertsen P (1989). Trial of intravenous lidocaine on painful neuropathy in cancer patients. Clin J Pain.

[15] Sharma S, Rajagopal MR, Palat G, Singh C, Haji AG, Jain D (2009). A phase II pilot study to evaluate use of intravenous lidocaine for opioid-refractory pain in cancer patients. J Pain Symptom Manage.

[16] Thomas J, Kronenberg R, Cox MC, Naco GC, Wallace M, von Gunten CF (2004). Intravenous lidocaine relieves severe pain: results of an inpatient hospice chart review. J Palliat Med.

[17] Fédération Nationale des Centres de Lutte Contre le Cancer, Standards, Options et Recommandations (2002). Standards, options et recommandations 2002 sur les traitements antalgiques médicamenteux des douleurs cancéreuses par excès de nociception chez l’adulte: mise à jour 2002.

[18] Bouhassira D, Attal N, Fermanian J, Alchaar H, Gautron M, Masquelier E, Rostaing S, Lanteri-Minet M, Collin E, Grisart J, Boureau F (2004). Development and validation of the neuropathic pain symptom inventory. Pain.

[19] Guirimand F, Buyck JF, Lauwers-Allot E, Revnik J, Kerguen T, Aegerter P, Brasseur L, Cleeland CS (2010). Cancer-related symptom assessment in France: validation of the French M. D. Anderson symptom inventory. J Pain Symptom Manage.

[20] Cleeland CS, Mendoza TR, Wang XS, Chou C, Harle MT, Engstrom MC (2000). Assessing symptom distress in cancer patients: the M.D. Anderson symptom inventory. Cancer.

[21] Clark ME, Gironda RJ, Young RW (2003). Development and validation of the pain outcomes questionnaire-VA. J Rehabil Res Dev.

[22] Evans CJ, Trudeau E, Mertzanis P, Marquis P, Pena BM, Wong J, Mayne T (2004). Development and validation of the pain treatment satisfaction scale (PTSS): a patient satisfaction questionnaire for use in patients with chronic or acute pain. Pain.

[23] Senn SJ (2002). Cross-Over Trials in Clinical Research.

[24] Agence Française de sécurité sanitaire des produits de santé (2011). Recommandations de bonne pratique: douleur rebelle en situation palliative avancée chez l’adulte. Médecine Palliative.

[25] Thorne FM, Morley S (2009). Prospective judgments of acceptable outcomes for pain, interference and activity: patient-determined outcome criteria. Pain.

